# Cytokine changes in cerebrospinal fluid following vascular surgery on the thoracic aorta

**DOI:** 10.1038/s41598-022-16882-0

**Published:** 2022-07-27

**Authors:** Christopher Pereira, Anisha H. Perera, Nung Rudarakanchana, Benjamin H. L. Harris, Matteo Di Giovannantonio, Simon D. Taylor-Robinson, Melanie Dani, Michael Fertleman

**Affiliations:** 1 Cutrale Perioperative and Ageing Group, Department of Bioengineering, Imperial College London, London, UK; 2 Imperial Vascular Unit, Department of Surgery and Cancer, Imperial College London, London, UK; 3 Computational Biology and Integrative Genomics, Department of Oncology, University of Oxford, Oxford, UK; 4 Department of Surgery and Cancer, Imperial College London, London, UK

**Keywords:** Cytokines, Blood-brain barrier

## Abstract

There is growing evidence that surgery can drive an inflammatory response in the brain. However, the mechanisms behind this response are incompletely understood. Here, we investigate the hypotheses that 1. Cerebrospinal fluid (CSF) cytokines increase after vascular surgery and 2. That these changes in CSF cytokines are interrelated. Patients undergoing either open or endovascular elective surgery of the thoracic aorta were invited to participate in this study. Cerebrospinal fluid samples were taken before surgery and on the first post-operative day. These were analysed for the presence of ten cytokines by immunoassay to examine for post-operative changes in cytokine levels. After surgery, there were significant increases in six out of the ten measured CSF cytokines (IL-1β, 2, 6, 8, 10 and 13). This included changes in both putative pro-inflammatory (IL-1β, 6 and 8) and putative anti-inflammatory (IL-2, 10 and 13) cytokines. The greatest increases occurred in IL-6 and IL-8, which showed a 63-fold and a 31-fold increase respectively. There was strong intercorrelation between CSF cytokines after the operation. Following surgery on the thoracic aorta, there was a marked increase in CSF cytokines, consistent with a potential role in neuroinflammation. The ten measured cytokines showed intercorrelation after the operation, indicating that a balance between multiple pro- and anti-inflammatory cytokines may be present.

## Introduction

Neuroinflammation is a complex and incompletely understood process which is partly mediated through cytokines^1^. It has been argued that the cytokine mediated neuroinflammatory process could drive peri-operative neurocognitive disorders (PND), such as post-operative delirium (POD), however this is a putative idea which has yet to be proven^2^. PND is the commonest post-operative complication in older patients undergoing surgery and is associated with worse clinical outcomes^3^; understanding its pathophysiology is essential for developing prevention and cure.

One hypothesis for the aetiology of PND is that a physiological insult from surgery induces the release of local inflammatory mediators (such as cytokines) from macrophages at the surgical site^4^. An intact blood–brain-barrier (BBB) prevents these inflammatory mediators from affecting the closely regulated environment of the CNS. However if the BBB is weakened by aging, neurodegeneration^5^, or the response to a surgical insult^6^, inflammatory mediators can enter the CNS. The processes by which the BBB may become weakened are complex, but in Alzheimer’s disease (AD), this includes brain capillary leakage, cellular infiltration, degeneration of pericytes and loss of endothelial integrity^5^. Once inflammatory mediators enter the CNS, microglial activation occurs, which drives further inflammation, partly through cytokine release^7,8^. Cytokine release and the inflammatory response can then trigger neuronal injury and dysfunction ^5^, which theoretically could manifest as PND. There is currently no treatment for PND^9^. If the process of neuroinflammation can be better understood, this may open the door to cytokine manipulation, potentially reducing levels of consequent PND.

Previous investigations on peri-operative cytokine changes has shown greater increases in CSF cytokines than serum or plasma cytokines after an operation^10–12^. This suggests a key role for CSF cytokines in post-operative neuroinflammation. However, the number of patients involved in these studies was small. There is a further suggestion that the CSF cytokines, IL-6 and IL-8, are increased in patients with worse neurocognitive outcomes, but again the sample sizes of these studies are inadequately powered to provide definitive evidence^11,13^.

To date, only one study has investigated peri-operative changes in both CSF and serum following vascular surgery on the thoracic aorta^14^. In this study of 23 patients, the cytokine IL-6 was increased in both serum and CSF after the operation. In patients with spinal cord injury, the CSF IL-6 increase was more marked. While this study examined a wide variety of proteins relevant to neurology, the number of cytokines studied was small.

Spinal cord injury leading to paraplegia is the most devastating consequence of morbidity following surgery on the thoracic aorta^15^. Draining CSF intraoperatively with a spinal catheter has been shown to reduce the chances of this complication^16^, by increasing blood flow to the spinal cord^17^. As such patients already have a spinal catheter in place. This allows for easy access to CSF peri-operatively, making patients undergoing thoracic-vascular surgery a valuable cohort for studying neuroinflammation.

We investigated cytokines with both a pro-inflammatory role (IL-1β, IL-6, IL-8, IL-12p70, IFN-γ and TNF-α) and those with an anti-inflammatory role (IL-2, IL-4, IL-10 and IL-13)^18,19^ in a cohort of patients undergoing vascular surgery. The aim of this study was to evaluate the neuroinflammatory response to vascular surgery in order to:

1. Determine changes in pro- and anti-inflammatory CSF cytokines before and after vascular surgery.
2. Investigate potential intercorrelations between cytokines in the CSF, to establish whether multiple cytokines are involved in driving neuroinflammation.
3. Establish if the changes in CSF cytokines are reflected in serum cytokine levels. If so, this would enable serum cytokines to be used as biomarkers of CSF changes.

## Methods

This was a prospective observational study looking at peri-operative changes in cytokine levels in the CSF and serum of patients undergoing elective vascular surgery for any pathology of the thoracic aorta.

### Study population

Patients were eligible for recruitment into the study if they were undergoing either open or endovascular repair for any thoracic aortic pathology in which a spinal catheter was to be used as part of standard peri-operative care. The decision about whether a spinal catheter would be used during an operation was a joint decision made pre-operatively in the vascular multidisciplinary team meeting involving the operating surgeon and anaesthetist. Patients did not have neuroimaging performed before or after the operation.

Recruitment took place at St Mary’s Hospital, London, over a one-year period. Patients unable to give informed written consent were excluded from the study. The study received approval from the London Westminster Research Ethic Committee (13/LO/0210). The study conformed to the precepts set out in the Declaration of Helsinki of 1975. All patients gave informed, written consent prior to surgery.

### Specimen collection and storage

Study patients had a spinal catheter inserted prior to surgery by an anaesthetist, following administration of a general anaesthetic. The spinal catheter was used to maintain the CSF pressure at 10 mmHg. If the pressure rose above 10 mmHg, CSF was slowly drained until the target was met. CSF was collected by the same operator (author AP) prior to surgery and on the first post-operative day. Approximately 1-3 mL of CSF was collected each time. The spinal catheter was removed when it was no longer clinically indicated. Samples were centrifuged at 4000RPM for 5 min to remove any red blood cells present in the sample. Samples were then pipetted into cryo-tubes in 500 μL aliquots and stored in a −80 °C freezer.

### Immunoassays

The V-PLEX Proinflammatory Panel 1 Human Kit (Meso Scale Discovery, Maryland, USA) was chosen as it measures both pro-inflammatory (IL-1β, IL-6, IL-8, IL-12p70, IFN-γ and TNF-α) and anti-inflammatory (IL-2, IL-4, IL-10 and IL-13) cytokines^19^. Analysis followed the manufacturer’s instructions (www.mesoscale.com). This method of cytokine measurement in peri-operative CSF and blood samples has been used previously^10^. It uses electrochemiluminescence to quantify the levels of ten cytokines in 25 μL of peri-operative CSF or blood samples.

The range for the lowest level of detection (LLD) was between 0.00861 pg/mL for IL-4 and 0.605 pg/mL for IFN-γ. If samples were above the upper limit of detection (ULD) for the assay, they were diluted in Diluent 2 which was supplied with the kit assay and re-analysed, according to the manufacturer’s instructions. This was necessary for a small number of IL-8 measurements. For cytokine levels below the LLD or detected, but below the fit curve, the LLD value for the cytokine assay was used, as previously described^20^. All cytokine analysis was completed in the Infectious Diseases Laboratory at Imperial College London, London.

### Statistical analysis

Analyses were carried out using Python, version 3.7 (available from www.python.org). A Wilcoxon Signed-Rank test with Bonferroni correction was used to examine changes in cytokines before and after surgery. Spearman’s rank correlation coefficient, again with a Bonferroni correction, assessed cytokine intercorrelation. An adjusted-p value of < 0.05 was considered statistically significant. Patient demographics (age and sex) were assessed for correlation with CSF cytokine changes using Spearman’s rank correlation coefficient with a Bonferroni correction and logistic regression respectively. A power calculation to determine the necessary sample size was not undertaken before starting the study, as the number of patients who would be able to consent and complete the study was anticipated to be small.

### Ethics approval and consent to participate

The study received approval from the London Westminster Research Ethic Committee (13/LO/0210). The study conformed to the precepts set out in the Declaration of Helsinki of 1975. All patients gave informed, written consent prior to surgery.

## Results

During a 1-year period, ten patients were recruited into the study. Table 1 shows the demographic and pre-operative information for the patients. Four of the patients were female, with a mean age of 65 yrs (SD 12 years). Seven patients had undergone previous vascular surgery, often with serious post-operative morbidity. Two patients died in critical care within 30-days of the operation. No patient developed post-operative paraplegia or a clinically apparent stroke.

**Table 1:** Demographic and Pre-operative Information.

ID	Sex	Age	Past Medical History	Past Surgical History	Drug History
4	F	73	HTN, high cholesterol, ex-smoker	Carotid-subclavian bypass	Bisoprolol, paracetamol, simvastatin
5	M	80	CABG, high cholesterol, prostate cancer	Radical prostatectomy, EVAR	Aspirin, simvastatin, omeprazole, paracetamol
10	F	56	Endometriosis, HTN, high cholesterol, ex-smoker	Hybrid-vascular surgery (2008)	Atorvastatin, amlodipine, perindopril
12	F	77	Left occipital ischaemic stroke, asthma, IHD, T2DM, high cholesterol, HTN	Nil	NR
13	F	40	HTN, Marfan syndrome with pectus excavatum and dural ectasia, heart failure	Type A aortic dissection—emergency aortic valve sparing open replacement of root/ascending aorta and proximal arch (2007), arch hybrid (2012)- post operative cardiac arrest followed by tracheostomy, myopathy and prolonged ITU stay	Amlodipine, perindopril, bisoprolol, aspirin, spironolactone
18	M	54	Marfan syndrome, ICD, blind, bilateral cataracts, stroke, HTN, high cholesterol, T2DM	Open arch and DTA replacement, AVR, and ascending aorta to LCCA bypass, post operative occipital infarct, haemorrhage and large SDH requiring craniotomy and evacuation	Amlodipine, indapamide, metoprolol, senna, doxazosin, gliclazide, losartan, pregabalin, lansoprazole, paracetamol, warfarin, tinzaparin
20	M	74	Paraplegia, CKD	Open type 2 thoracic aorta repair (2000), then dilatation of visceral aortic patch	NR
24	M	62	HTN	Nil	Carvedilol
25	M	72	HTN, high cholesterol, current smoker, T2DM, COPD	Nil	NR
27	M	65	HTN, high cholesterol, smoker, COPD, schizophrenia	EVAR (2013)	Amlodipine, procyclidine

*AVR* Aortic valve replacement, *CKD* Chronic kidney disease, *CABG* Coronary artery bypass graft, *COPD* Chronic obstructive pulmonary disease, *DTA* Descending thoracic aorta, *EVAR* Endovascular aneurysm repair, *HTN* Hypertension, *ICD* Implantable Cardioverter Defibrillator, *IHD* Ischaemic heart disease, *ITU* Intensive therapy unit, *LCCA* left common carotid artery, *NR* Not recorded, *SH*D Subdural haematoma, T2DM Type 2 Diabetes. A table showing the medical and surgical backgrounds for the ten patients included in this study.

### Timetable of sample collection

All patients had pre-operative (timepoint 1: T1) and post-operative (timepoint 2: T2) CSF samples taken. Five patients also had simultaneous paired serum samples. Patient 5 had their post-operative samples taken on the day of surgery. Supplementary table 1 summarises the aetiology of the thoracic aorta pathology, the type of vascular surgery and the post-operative complications.

### Cytokine changes following surgery

**Figure 1:**
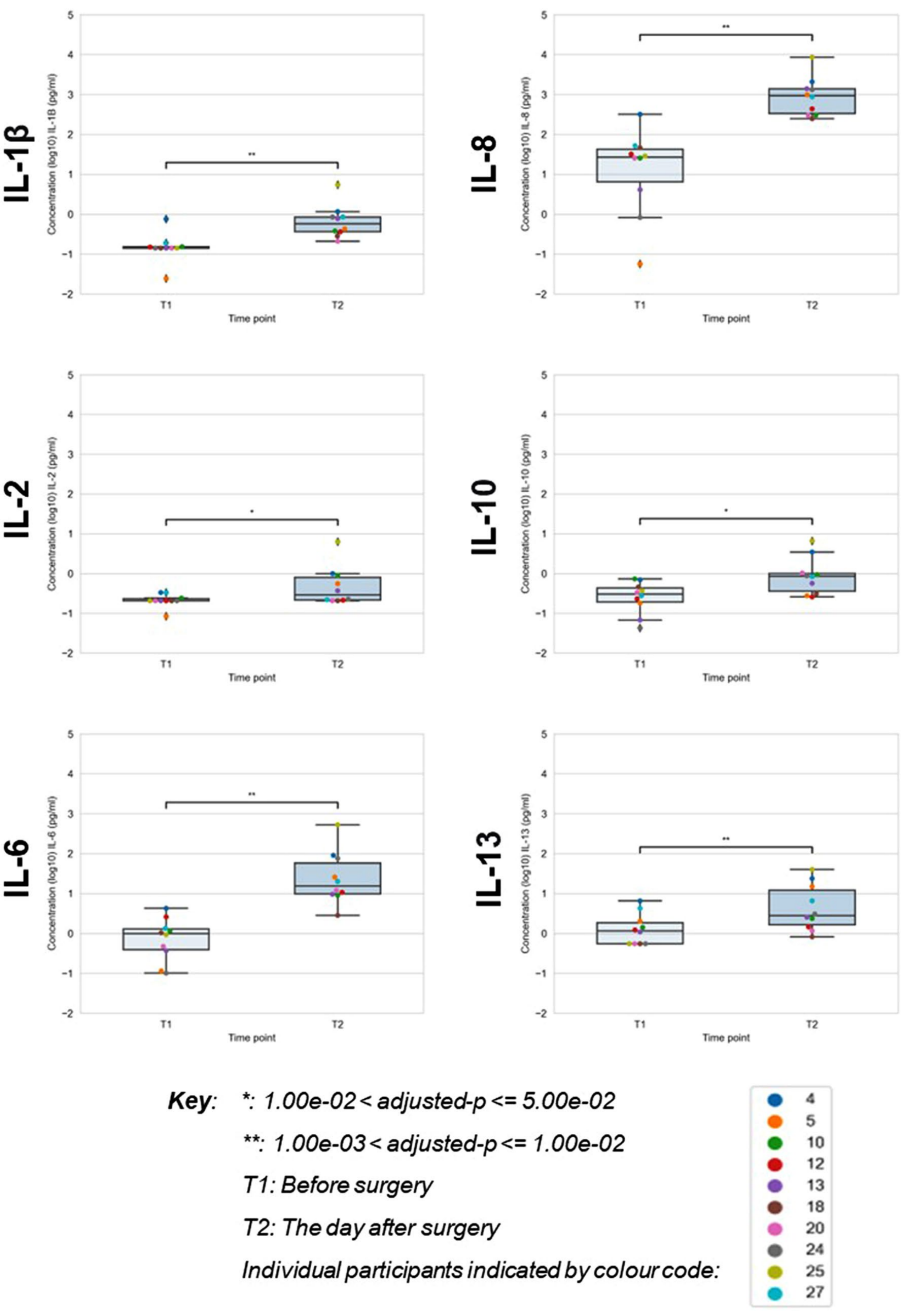
Post-operative changes in CSF cytokines. Levels of cytokines in cerebrospinal fluid (CSF) before surgery (T1) and the day after surgery (T2). Each patient is represented by a colour that is consistent across the box-plots. Only cytokines that showed statistically significant changes following surgery are shown.

Table 2 and Fig. 1 show the cytokine changes in CSF following surgery. The greatest increases occurred in IL-6 and IL-8, which showed a 63-fold and a 31-fold increase respectively. Six cytokines showed statistically significant increases between T1 and T2 (IL-1β, 2, 6, 8, 10 and 13), as shown in Fig. 1. Patient demographics (age and sex) were not significantly correlated with any CSF cytokine changes. Of the cytokines which showed statistically significant increases, three are traditionally classified as pro-inflammatory (IL-1β, 6 and 8) and three (IL-2, 10 and 13) as anti-inflammatory^18^.

**Table 2:** Summary of cytokine measurements in CSF.

Cytokine	Timepoint	CSF Median [IQR] (pg/mL)	Adjusted-*p* value Wilcoxon Signed-Rank Test between T1 and T2	Average fold change
IL-1β	T1 T2	0.14 [0.14–0.15] 0.60 [0.36–0.84]	< 0.01	5.4
IL-2	T1 T2	0.20 [0.20–0.23] 0.30 [0.21–0.81]	< 0.05	4.6
IL-4	T1 T2	0.02 [0.01–0.03] 0.03 [0.01–0.08]	ns	4.5
IL-6	T1 T2	0.98 [0.39–1.28] 15.91 [9.73–62.57]	< 0.01	63.3
IL-8	T1 T2	26.85 [9.35–42.32] 919.23 [333.42–1366.63]	< 0.01	31.0
IL-10	T1 T2	0.30 [0.19–0.43] 0.85 [0.37–1.00]	< 0.05	4.5
IL-12p70	T1 T2	0.09 [0.08–0.17] 0.11 [0.08–0.12]	ns	2.8
IL-13	T1 T2	1.14 [0.55–1.86] 2.78 [1.67–12.75]	< 0.01	5.2
IFN-γ	T1 T2	0.605 [0.54–0.67] 0.605 [0.54–0.63]	ns	2.6
TNF-α	T1 T2	0.52 [0.32–0.96] 0.99 [0.51–2.10]	ns	3.9

Cytokine levels in cerebrospinal fluid (CSF) before surgery (T1) and the day after surgery (T2), ns = non-significant.

The levels of most CSF cytokines tended to rise following surgery. However, levels of IFN-γ and IL-12p70 were generally below the LLD in both the pre-and post-operative CSF samples, so no change could be detected. For the cytokines IL-1β, IL-2 and IL-4, pre-operative levels were often below the LLD, but post-operative levels rose into the detection range.

### Intercorrelation between CSF cytokines

**Figure 2:**
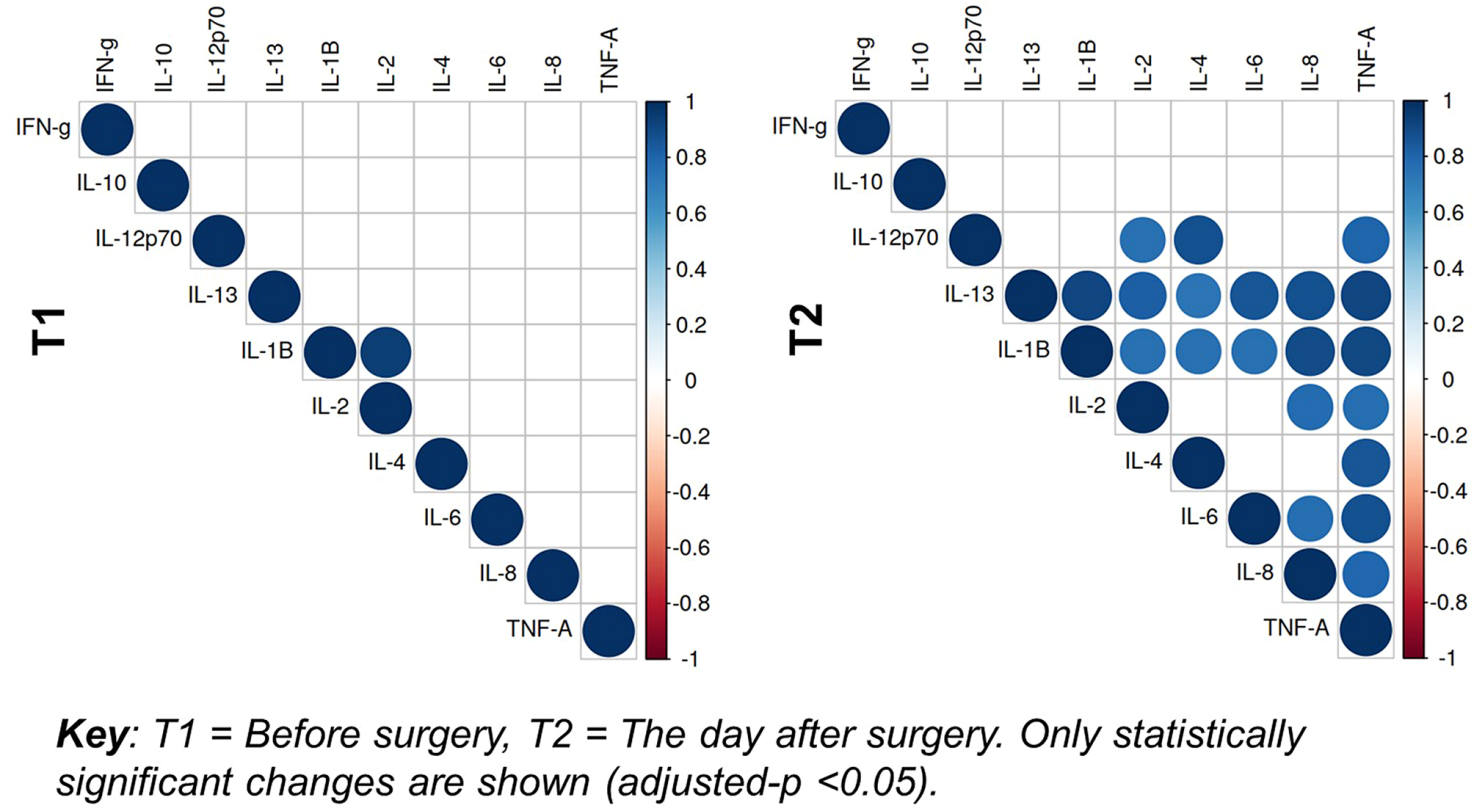
Correlation plots between CSF cytokines before and after surgery. Correlation plots between cytokines for cerebrospinal fluid (CSF) before surgery (T1) and the day after surgery (T2).

Figure 2 shows the intercorrelation between CSF cytokines before and after surgery. At T1, the significant correlations observed in the CSF were between IL-1β and IL-2 (r = 0.95, adjusted-*p* < 0.001). At T2, there were a number of strong positive correlations between 20 pairs of different cytokines, as shown in Supplementary table 2.

### Serum cytokine changes

Only five of the ten patients had serum samples drawn at the same time as the CSF samples. The greatest increase was in IL-6 which showed a 71-fold increase in serum levels after the operation. There was limited intercorrelation between CSF and serum cytokines. At T1, four combinations of CSF and serum cytokines showed significant correlation (r = 1, adjusted-*p* < 0.05). These included IL-2 and IL-1β, IL-4 and IL-12, IFN-γ and Il-2 and IL-1β and IL-1β. At T2 there were no significant correlations between the CSF and serum cytokine levels (data not shown).

## Discussion

### CSF cytokine findings

We demonstrated increases in both pro- and anti-inflammatory CSF cytokines after vascular surgery on the thoracic aorta. The most marked increases occurred in the pro-inflammatory cytokines, IL-6 and IL-8. The increases in CSF IL-6 confirm previous findings in a similar cohort^14^, whereas, to our knowledge, we are the first group to show similar changes in CSF IL-8. Interleukin-8 is a member of the CXC chemokine family, implicated in a wide variety of inflammatory diseases^21^. CSF IL-8 has been shown to be increased in AD, Parkinson’s disease^22^ and following traumatic brain injury^23^. In patients undergoing other forms of surgery, findings of marked increases in CSF IL-8 have also been demonstrated^10,12^.

The increase in CSF cytokines in this study could have resulted from a dysregulated inflammatory response to the peripheral stimulus of surgery driving neuroinflammation within the brain. Alternatively, these findings may have been secondary to silent cerebral infarcts, which have been shown to be increased following thoracic aortic endovascular procedures^24^. Ischaemic strokes have been shown to drive an increase in proinflammatory cytokines^25^. It is also possible that the CSF cytokine changes may have occurred due to other post-operative complications such as endoleaks or infection.

### Intercorrelation findings

Levels of cytokines in the CSF, while not exhibiting an association with each other prior to surgery, strongly correlate on day one after surgery. Figure 2 highlights the complex balance between pro- and anti-inflammatory cytokines which may drive neuroinflammation. The lack of an association between post-operative CSF and serum samples is consistent with other studies in this area^10,12^, and suggests that changes in cytokine levels in blood cannot be used reliably as surrogate markers of CSF cytokine changes.

### Proposed mechanisms

The results of this study need to be interpreted alongside the current postulated mechanisms of brain dysfunction. These include excessive neuroinflammation^26^, the production of reactive oxygen species (ROS)^27^, and dysregulated neurotransmission^28^.

These mechanisms are all mediated through activation of microglia, which when stimulated can release ROS^27^ and cytokines^29^. Microglia are also key drivers of the kynurenine inflammatory pathway, which, in turn, can drive glutamatergic neurotransmission^30^. In the healthy brain, microglia are fundamental in maintaining tissue homeostasis by removing accumulated debris^27^. However, their overactivity may be harmful in disease states^31^.

The postulated mechanisms of brain dysfunction are not mutually exclusive, with multiple mechanisms likely to be acting together^28^. Indeed, pro-inflammatory cytokines have been shown to activate the kynurenine pathway, which in turn leads to the generation of ROS through quinolinic acid production^32^. The cytokine changes demonstrated in this study may therefore have implications for several mechanisms of brain dysfunction.

### Therapeutic targets

In contrast to neurodegenerative processes, peri-operative brain dysfunction occurs at a predictable time point, giving a potential opportunity for prevention^33^. Currently, no effective treatment for PND exists^34^. The suggestion that CSF IL-6 and IL-8 hold a key role in the post-operative neuroinflammatory pathway, raises the question of whether direct cytokine inhibition could attenuate these effects. However, as we have demonstrated in this paper, multiple cytokines, both pro- and anti-inflammatory, increase after an operation and thus blocking the action of only one of these cytokines may not necessarily inhibit neuroinflammation. Furthermore, as has been suggested in Alzheimer’s disease, a degree of cytokine-driven neuroinflammation may be neuroprotective^35,36^.

### Limitations

Similar to many studies in this area, this study had a small patient cohort, which may have limited our ability to demonstrate the true magnitude of peri-operative cytokine changes. Future studies should involve larger sample sizes across multiple settings to address this problem. Samples were only taken at two time points, with one patient’s CSF sample taken after surgery on day 0 rather than day 1. This patient was not excluded due to an already small cohort. Within the cohort, the surgical approach was not homogenous, with some patients undergoing open surgery and others undergoing endovascular surgery, which could represent a further complicating factor. The small numbers of patients in different surgical groups limits meaningful comparisons. A further confounder was that seven out of ten patients had undergone previous vascular surgery, often with serious post-operative morbidity. Ideally, this study would have corrected for underlying co-morbidity and baseline inflammatory status. In future studies, neuroimaging, to look for radiological evidence of stroke, should be included to investigate how much of the neuroinflammatory burden may be driven by ischaemic strokes. Finally, we cannot exclude the possibility that the inflammatory response was driven by the insertion of the spinal catheter, rather than surgery or anaesthesia, but this is felt to be unlikely due to the magnitude of cytokine changes^12^.

This study looked solely at cytokine changes, which is only part of the neuroinflammatory process after surgery^33^. Measurement of the Q-albumin to determine the integrity of the BBB would also have been useful^5^. Future studies would ideally also examine the CSF cell count and immunoglobulin subtypes^37^, and other markers of neuronal injury^6^ to more fully understand pathophysiological processes. This was not possible within the scope of this study.

A final key limitation of this study was that patients did not undergo peri-operative cognitive testing. Formal cognitive testing for delirium, using screening tools such as the 4AT^38^ and neuropsychological testing, would allow for the more direct investigation of correlations between observed CSF cytokine changes and the magnitude of cognitive dysfunction in PND.

## Conclusions

After vascular surgery there is a large increase in cytokines in the CSF, particularly in the pro-inflammatory cytokines IL-6 and IL-8. This may be secondary to peripheral changes in the circulation crossing the BBB and driving neuroinflammation. A strong correlation was found between CSF cytokines on day one after the operation, suggesting that it may be the balance between multiple pro- and anti-inflammatory cytokines which drives neuroinflammation.

## Supplementary Information

Supplementary Information.

## Data Availability

The dataset supporting the conclusions of this article is available from the corresponding author upon reasonable request.

## Abbreviations

AD: Alzheimer’s disease
BBB: Blood–brain barrier
CNS: Central nervous system
CSF: Cerebrospinal fluid
IFN-γ: Interferon gamma
IL: Interleukin
LLD: Lower limit of detection
POD: Post-operative delirium
PND: Peri-operative neurocognitive disorders
ROS: Reactive oxygen species
T1: Time-point 1 (pre-operation)
T2: Time-point 2 (day 1 post-operation)
TEVAR: Thoracic endovascular aortic repair
TNF-α: Tumour necrosis factor alpha
ULD: Upper limit of detection
