# Supplementary material for: Cytokine changes in cerebrospinal fluid following vascular surgery on the thoracic aorta
Source: Sci Rep. 2022 Jul 27;12:12839. doi: 10.1038/s41598-022-16882-0 (PMC9329310; doi:10.1038/s41598-022-16882-0)
Supplement: Supplementary file 1 — Supplementary Information. [file 41598_2022_16882_MOESM1_ESM.pdf]

Supplementary material for the manuscript 'Cytokine Changes in Cerebrospinal Fluid Following Vascular Surgery on the Thoracic Aorta'

Supplementary Table 1: Operation type and post-operative events

ID	Aetiology	Aneurysm type	Operation	LOS (days)	Post-operative complications
4	Atherosclerosis	2	TEVAR	N/A	Died in ITU after the operation (day 3)
5	Traumatic transection 6 months before recruitment	DTA	TEVAR	3	Nil
10	Atherosclerosis	3	TEVAR	12	Post-TEVAR laparotomy for bleeding due to iliac rupture and left to right ilio-femoral cross over. HAP
12	Atherosclerosis	DTA	TEVAR	NR	Endoleak requiring TEVAR extension
13	Chronic type B dissection	2	TEVAR + open revascularisation visceral and renal vessels	24	HAP
18	Chronic type B dissection + aneurysm	NR	TEVAR extension	7	Endoleak
20	Atherosclerosis	2	TEVAR + open revascularisation visceral and renal vessels	N/A	Died in ITU after the operation
24	Atherosclerosis	3	Open repair	18	AF
25	Chronic type B dissection + aneurysm	3	Open repair	NR	Post-operative tracheostomy, VAP, pleural effusions, ileus
27	PAU	Acute PAU	TEVAR + left carotid-subclavian bypass	12	AKI, HAP

Key: AF = Atrial fibrillation, AKI = Acute kidney injury, DTA = Descending thoracic aortic, HAP = Hospital-acquired pneumonia, ITU = Intensive therapy unit, LOS = Length of stay, NR =Not recorded, N/A = Not applicable, PAU = Penetrating atherosclerotic ulcer, TEVAR = Thoracic endovascular aortic repair, VAP = Ventilator associated pneumonia

Supplementary Table 2: Intercorrelation between CSF cytokines at T2 (day 1 post-operation)

Intercorrelation between cytokines	Adjusted-p	R value
IL-1β and IL-2	<0.05	0.75
IL-1β and IL-4	<0.05	0.75
IL-1β and IL-6	<0.05	0.76
IL-1β and IL-8	<0.01	0.89
IL-1β and TNF-α	<0.01	0.90
IL-2 and IL-8	<0.05	0.78
IL-2 and TNF-α	<0.05	0.77
IL-4 and TNF-α	<0.01	0.85
IL-6 and IL-8	<0.05	0.77
IL-6 and TNF-α	<0.01	0.88
IL-8 and TNF-α	<0.05	0.79
IL-12p70 and IL-2	<0.05	0.83
IL-12p70 and IL-4	<0.01	0.88
IL-12p70 and TNF-α	<0.05	0.80
IL-13 and IL-1β	<0.01	0.92
IL-13 and IL-2	<0.05	0.83
IL-13 and IL-4	<0.05	0.73
IL-13 and IL-6	<0.01	0.85
IL-13 and IL-8	<0.01	0.88
IL-13 and TNF-α	<0.01	0.92

Key: Only statistically significant changes shown (adjusted-p <0.05)

Legend: A table showing the statistically significant intercorrelation findings between cerebrospinal fluid (CSF) cytokines at T2 (day 1 post-operation).
